# *Para*-Cresol and the Brain: Emerging Role in Neurodevelopmental and Neurodegenerative Disorders and Therapeutic Perspectives

**DOI:** 10.1021/acsptsci.5c00289

**Published:** 2025-09-15

**Authors:** Laura Bertarini, Federico Imbeni, Virginia Brighenti, Isabella Martusciello, Federica Pellati, Silvia Alboni

**Affiliations:** † Department of Life Sciences, 9306 University of Modena and Reggio Emilia, Via Giuseppe Campi 103-287, 41125 Modena, Italy; ‡ Clinical and Experimental Medicine PhD Program, Department of Biomedical, Metabolic and Neural Sciences, University of Modena and Reggio Emilia, Via Giuseppe Campi 287, 41125 Modena, Italy; § Center for Neuroscience and Neurotechnology, University of Modena and Reggio Emilia, 41121 Modena, Italy

**Keywords:** *p*-cresol, autism, Alzheimer’s disease, Parkinson’s disease, stress, brain

## Abstract

*p*-Cresol (*p*C) is a phenolic compound to which humans can be exposed through both environmental sources, such as a pollutant, and endogenous production by the gut microbiota. Among microbial contributors, *Clostridioides difficile* appears to be a major source of *p*C within the body. Once absorbed, *p*C is highly protein-bound in plasma and predominantly circulates in its hepatic conjugated forms: *p*-cresyl sulfate (*p*CS) and *p*-cresol glucuronide (*p*CG), which are mainly excreted in urine. Accumulation of these metabolites, particularly *p*CS, classified as a protein-bound uremic toxin, has been associated with the progression of chronic kidney disease (CKD) and related complications, due to its pro-oxidant, pro-inflammatory, and pro-apoptotic properties. CKD patients are at increased risk for cognitive impairment, affective disorders, and central nervous system (CNS) dysfunctions. In recent years, increasing evidence has suggested a potential role of *p*C and its metabolites in CNS diseases. Here, we summarize current knowledge on the involvement of these compounds in the pathogenesis and progression of autism spectrum disorder, Parkinson’s disease, Alzheimer’s disease, and post-traumatic stress disorder. We also discuss how modulating systemic levels of *p*C may represent a promising strategy to improve pathological phenotypes in the context of neurodevelopmental and neurodegenerative disorders.

## Graphical abstract

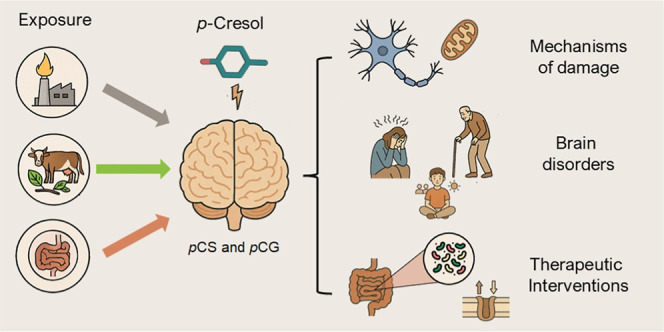

*p*-Cresol (4-methylphenol, *p*C) is a partially lipophilic compound (log *P* 1.94) with low-molecular-weight, belonging to the large family of phenols.^1^ The interest in the role of this compound related to the etiopathogenesis of central diseases, including those associated with neurodevelopment and neurodegeneration, has steadily increased in recent years. Comprehensive data on the general toxicology of *p*C, including its physicochemical properties, environmental distribution, uses, exposure routes, metabolism, and potential health effects, are available from several authoritative sources.^2,3^

The present review aims to provide comprehensive information on *p*C, its presence and origin in the human body, its pharmacokinetic properties, and its linkage with the pathogenesis of serious central nervous system (CNS) diseases, with regard to autism (ASD), Alzheimer’s Disease (AD), Parkinson’s Disease (PD), and Post Traumatic Stress Disorder (PTSD). Neurophysiological and neuropathological conditions involving *p*C are listed in Table 1, which will be thoroughly discussed in this review.

**Table 1:** Representative Neurophysiological and Neuropathological Conditions for Which an Involvement of the *p*-Cresol and its related metabolites Has Been Suggested

conditions	species	refs
Neurodevelopmental Disease		
Autism Spectrum Disorder (ASD)	*human*	^111^
ASD	*human*	^113^
ASD	*human*	^123^
ASD	*human*	^120^
ASD	*rat*	^124^
ASD	*human*	^121^
ASD	*human*	^95^
ASD	*mouse*	^96^
ASD	*mouse*	^62^
ASD	*mouse*	^60^
ASD	*human*	^116^
ASD	*mouse*	^94^
ASD	*human*	^63^
ASD	*human*	^114^
Neurodegenerative Disease		
Parkinson’s Disease (PD)	*human*	^154^
PD	*human*	^161^
PD	*human*	^151^
PD	*human*	^149^
PD	*human*	^127^
PD	*human*	^140^
PD	*human*	^150^
multiple sclerosis	*human*	^93^
Alzheimer’s Disease (AD)	*rat*	^180^
AD	*mouse*	^62^
AD	*human*	^187^
AD	*human*	^188^
Neuropsychiatric Disorder		
Post-Traumatic Stress Disorder (PTSD)	*mouse*	^94^
PTSD	*mouse*	^57^

## *p*-Cresol Exposure and Disposition: Insights into Its Absorption, Distribution, Metabolism, and Elimination

Given the complex pathways through which *p*C is absorbed, metabolized, distributed, and eliminated, Figure 1 provides a comprehensive overview of its ADME profile and related metabolites, including the main sources of exposure. This facilitates understanding of the multiple processes discussed throughout this section and emphasizes the predominant role of microbial production, first-pass metabolism, distribution, and primary routes of excretion.

**Figure 1:**
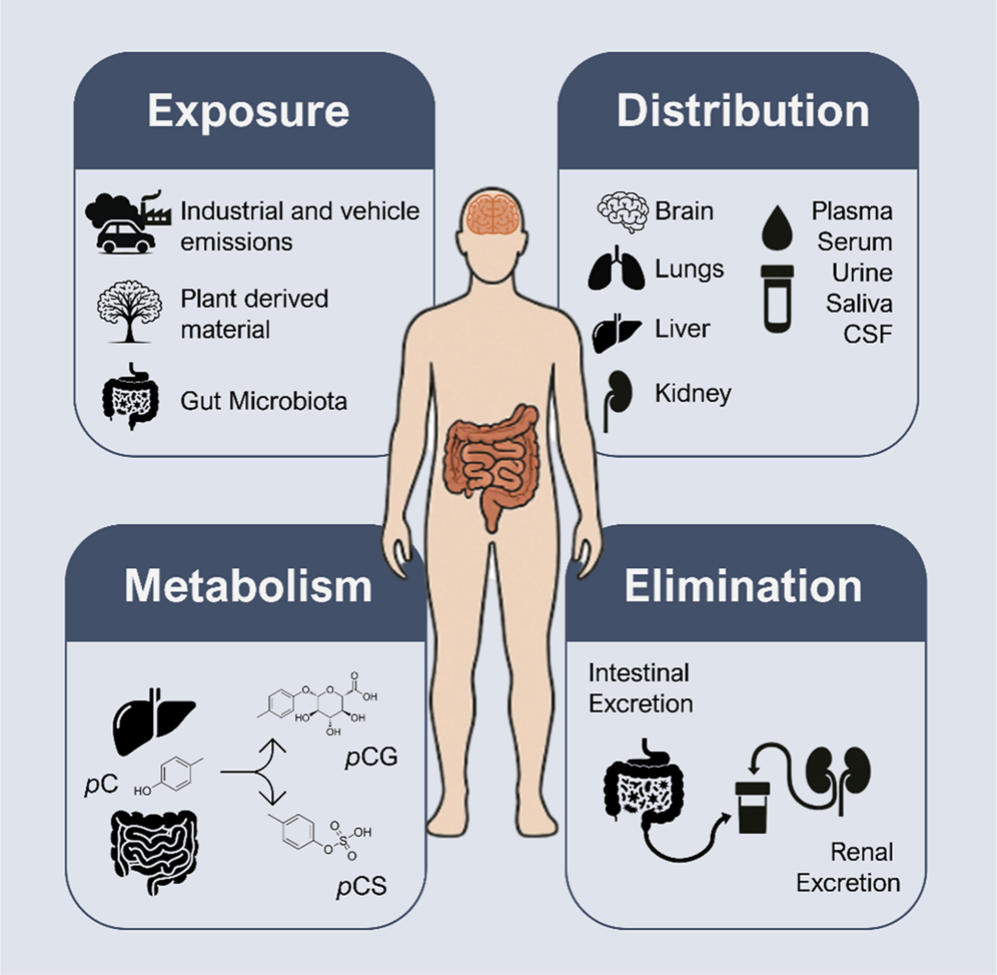
Absorption, distribution, metabolism, and excretion (ADME) of *p*C and its related metabolites. *p*C exposure occurs via inhalation, ingestion, dermal contact, and, mainly, microbial production in the gut. In the body, *p*C is absorbed in the intestine and primarily metabolized into *p*-cresyl sulfate (*p*CS) and glucuronide (*p*CG), with *p*CS being predominant, especially in uremic conditions. Distribution varies by tissues, with *p*CS prevailing in blood, kidneys, and lungs, and free *p*C enriched in the liver. Renal excretion is the main elimination route, with smaller contributions from feces and the lungs. The figure was created using icons from the Mind the Graph platform.

*p*C is widely distributed in the environment, originating from both natural and anthropogenic sources. Natural sources include microbial metabolism and plant material degradation, whereas anthropogenic contributions stem primarily from industrial activities, such as petrochemical processing, waste incineration, and vehicle emissions (Figure 1).^2–4^ Despite this environmental presence, evidence from germ-free animal models and antibiotic treatment studies indicates that the gut microbiota (GMB) is the primary endogenous source of *p*C.^5,6^ In the gut, phenolic compounds like *p*C are mainly produced through microbial metabolism of l-tyrosine and, to a lesser extent, l-phenylalanine.^7^ At least 55 bacterial species have been identified as *p*C producers, with *Blautia hydrogenotrophica*, *Clostridioides difficile*, *Olsenella uli*, and *Romboutsia ilealis* recognized as major contributors.^4,8^ Supporting the role of the GMB in *p*C production, antimicrobial treatments such as neomycin and vancomycin have been shown to suppress its endogenous synthesis.^5^ Furthermore, metabolomic studies provide additional evidence for the pivotal role of GMB in *p*C production, demonstrating that variations in microbial composition significantly affect systemic levels.^6^

Gut *p*C production is strongly influenced by dietary and physiological factors: high-protein diets promote the growth of proteolytic bacteria, leading to increased *p*C levels,^9^ while fiber-rich diets and probiotic supplementation reduce systemic concentrations by reshaping microbial communities.^10–13^ Fasting has also been shown to elevate *p*C levels in rats, likely due to reduced fiber intake, slower intestinal transit, and greater availability of endogenous proteins, which favor proteolytic activity and *p*C absorption.^14^ Additional factors, such as l-tyrosine intake and gastric acid suppression therapy, further influence *p*C production.^12,13,15,16^ Moreover, carbon source availability is a key regulator of *p*C production in the gut, with glucose promoting its synthesis and lactic acid inhibiting it.^17^

*p*C is produced through the fermentation of l-tyrosine via the intermediate *p*-hydroxyphenylacetate (*p*HPA), which is then decarboxylated by the glycyl radical enzyme 4-hydroxyphenylacetate decarboxylase (HpdBCA).^18^ The detection of *p*HPA in human colon at concentrations up to 19 μM^19^ supports its physiological relevance as a precursor for in vivo *p*C synthesis. Functional studies have demonstrated that disruption of any gene encoding the HpdBCA complex abolishes *p*C production in *C. difficile*.^20,21^

Following its biosynthesis, *p*C exerts antimicrobial effects against Gram-negative bacteria both *in vitro* and in inin vivo infection models, with *C. difficile* showing a markedly higher tolerance to *p*C than other common gut microbes, such as *Gammaproteobacteria* and *Bacteroidetes*, thus suggesting a competitive advantage within the intestinal niche.^20^

Once absorbed, *p*C undergoes extensive first-pass metabolism in the intestine and liver, i.e., conjugation in enterocytes and hepatocytes, primarily forming *p*-cresol glucuronide (*p*CG) and *p*-cresyl sulfate (*p*CS)^22,23^ (Figure 1). Glucuronidation of *p*C is primarily mediated by UDP-glucuronosyltransferase (UGT) enzymes, with UGT1A6 being the main isoform involved, responsible for over 78% of hepatic and over 54% of renal *p*C conjugation.^24^ UGT1A6 activity follows a sigmoidal kinetic model, indicating the presence of multiple substrate-binding sites.^25^ Conversely, UGT1A9 contributes to a lesser extent and exhibits substrate inhibition at high *p*C concentrations.^26,27^ Although these amounts are unlikely under physiological conditions, levels approaching 700 μM, considered potentially toxic,^28^ may impair UGT1A9 activity and limit *p*C detoxification via glucuronidation, possibly as a self-limiting or protective mechanism.

Sulfation of *p*C is primarily mediated by the SULT1A1 enzyme, which follows a Michaelis-Menten kinetic in the liver, but exhibits substrate inhibition in the kidneys, suggesting complex regulatory dynamics.^29^ Even if hepatic sulfation has long been considered the main route for producing *p*CS, emerging evidence highlights a significant contribution from the intestinal epithelium.^30,31^ Indeed, SULT1A1 is expressed not only in the liver but also in the small intestine and colon, where it catalyzes sulfation of phenolic compounds.^30^ This local intestinal expression aligns with the microbial origin of *p*C in the gut lumen, suggesting that sulfation may begin immediately after absorption. Indeed, transcriptomic and enzymatic studies demonstrate *p*C-induced upregulation of SULT1A1 in gut epithelial tissues, indicating a regulatory role of microbial metabolites on host detoxification enzymes.^32^

Due to extensive first-pass metabolism, the predominant circulating forms of *p*C in humans are its conjugated metabolites.^33^ Only a minor fraction of unconjugated *p*C remains in plasma, where it circulates predominantly in a protein-bound form.^34^ Albumin plays a key role in modulating plasma levels, acting as the main carrier for both *p*C and *p*CS,^34,35^ with more than 90% of the latter being in the protein-bound form.^22^ This has been further confirmed by ultrafiltration studies identifying both high-affinity and low-affinity binding sites.^36^ In contrast, *p*CG shows a lower binding affinity to albumin, with a larger fraction circulating in its free form.^37^ However, binding affinity values vary across studies: while some report strong interactions, others describe only weak binding between *p*C or *p*CS and albumin, as observed by Bergé-Lefranc *et al.* ^34^ These discrepancies may be influenced by environmental factors like temperature, which can affect protein binding.^34^ Notably, conditions such as hypoalbuminemia may alter the distribution of *p*C and its metabolites, increasing their unbound plasma fractions.^38^

In healthy individuals, unconjugated *p*C is present in blood at very low concentrations, typically around 0.4 nM, while the majority circulates in its conjugated forms as a result of efficient first-pass metabolism.^33,39^ In conditions of impaired renal function, such as chronic kidney disease (CKD) and uremia, blood and organ concentrations of *p*C, *p*CS, and *p*CG rise significantly, due to reduced renal clearance.^40,41^ For example, serum *p*CS increases from 15 to 35 μM in healthy subjects to 116–568 μM in uremic conditions.^22^ Despite variable and disproportionate patterns described in the literature,^40,42–45^ *p*CS shows the most consistent correlation with CKD progression.^46,47^ Given their predominance in circulation, *p*CS and *p*CG are hypothesized to mediate most of the biological effects in the body.^22,48^

The elimination of *p*C and its conjugated forms occurs predominantly by renal excretion, with urinary clearance playing a key role in maintaining systemic homeostasis^15,49,50^ (Figure 1). Among the conjugates, *p*CS is the principal urinary metabolite, actively secreted by renal tubular cells via organic anion transporters (OATs), particularly OAT1 and OAT3, which mediate its efficient clearance.^51^ In healthy subjects, about 95% of urinary *p*C is excreted as *p*CS and approximately 5% as *p*CG.^52^ Accordingly, reduced renal clearance in pathologies, such as CKD, may explain the increased accumulation of these compounds in both the blood and tissues. Another excretion route is through feces; indeed, *p*C and *p*CS have been detected in healthy individuals.^53,54^

Alongside uremic conditions, elevated concentrations of *p*C and its conjugated forms have been described in both neurodevelopmental and neurodegenerative disorders, including ASD, PD, AD, and PTSD.^55–58^ The consistent increase in these gut-derived metabolites in these conditions suggests they may not merely reflect microbial imbalance or impaired host detoxification but actively contribute to disease processes. Their augmented presence in plasma, urine, feces, and even cerebrospinal fluid (CSF) underscores their ability to reach the CNS and influence neurological outcomes.^55–58^ As such, these molecules may represent potential mediators in the pathophysiology of gut-brain axis disorders.

## Mechanisms of Damage Caused by *p*-Cresol and Metabolites

*p*C and its primary metabolites, particularly *p*CS, have been associated with multiple pathological mechanisms across different organ systems and models.^4,41,55,59–61^ These compounds are increasingly recognized as contributors to neurotoxicity and systemic inflammation, all of which may be relevant in the context of neurodevelopmental and neurodegenerative disorders.^55,59,62,63^

A summary of the main molecular pathways through which *p*C and its metabolites are implicated in mechanisms of toxicity and dysfunction is provided in Figure 2, along with an overview of the major disorders in which they are involved. The main molecular mechanisms underlying the *in vitro*/*in vivo* effects of *p*C and its metabolites are summarized in Table 2.

**Figure 2:**
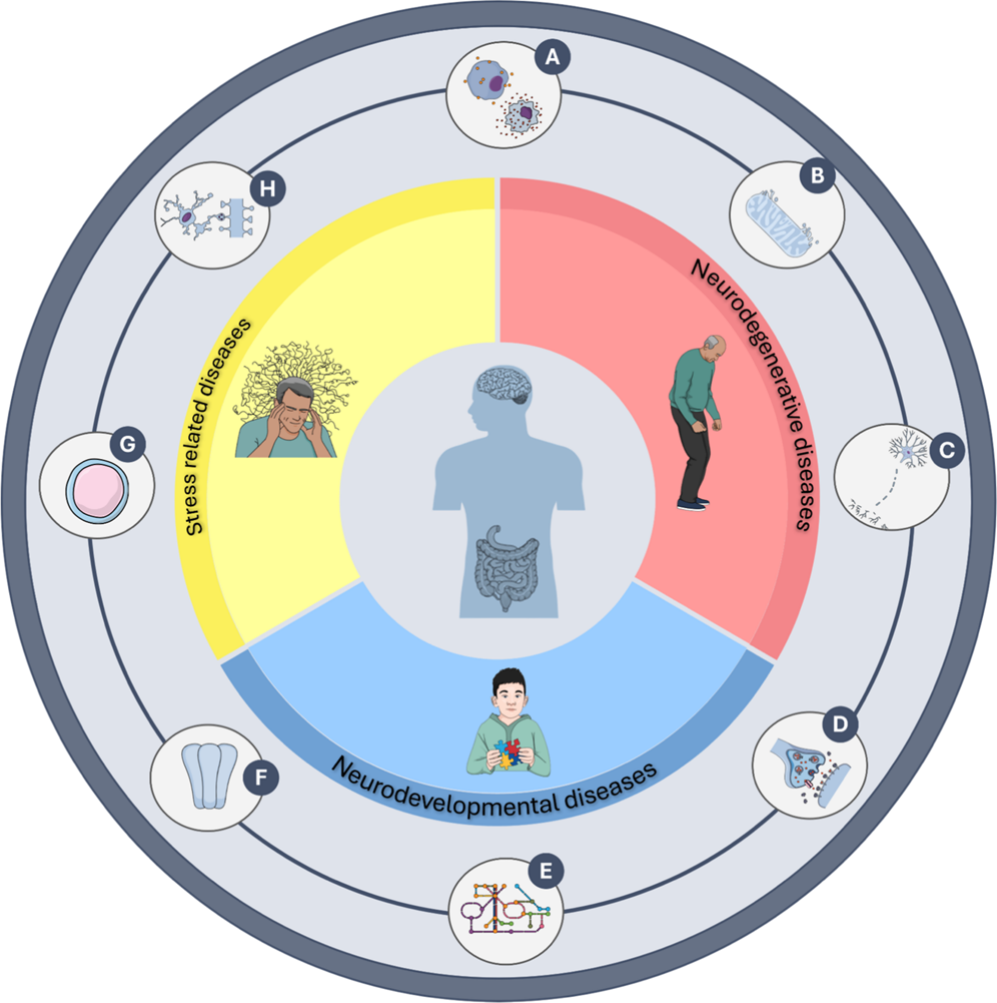
Schematic representation of the main effects of *p*C and its metabolites on the CNS. The diagram highlights the key neurotoxic mechanisms associated with *p*C and its metabolites, including: (A) oxidative stress: increased reactive oxygen species (ROS), GSH depletion, and imbalance between pro- and antioxidant systems; (B) mitochondrial dysfunction: impaired Krebs cycle, structural mitochondrial damage, and energy imbalance; (C) neuroinflammation and damage to neurons and oligodendrocytes: activation of inflammatory signaling pathways (e.g., NF-κB, JNK), elevated IL-1β levels, and reduced neuronal survival markers; (D) synaptic alterations: reduced synaptic density, impaired expression of key synaptic proteins, and dendritic remodeling deficits; (E) neurotransmitter metabolism dysfunction: DA accumulation due to DBH inhibition, 5-HT, and BDNF impairment; (F) receptor activity disruption: altered NMDAR or subunit composition, receptor mislocalization, and reduced Na^+^/K^+^-ATPase activity; (G) systemic immunosuppression: inhibition of Th1 immune responses, decreased IFN-γ, and shift toward a Th2 cytokine profile; and (H) microglial dysfunction and impaired brain immune responses: suppression of pro-inflammatory cytokine release (TNF-α, IL-6) and inhibition of ADAM10/17 activity. The figure was created using icons from the Mind the Graph platform.

**Table 2:** Main *In Vitro* and *In Vivo* Effects of *pC* and Its Metabolites^a^

experimental model	exposure	effect	refs
***In Vitro***			
hepatic tumor cell line (HepaRG)	*p*C	↑ DCF formation	^41^
		↑ lactate dehydrogenase (LDH) release	
		↓ GSH concentration	
human liver microsomes	*p*C	↑ cytochrome P450-mediated aromatic oxidation	^74^
mesenchymal stem cells (MSC)	*p*C	↑ apoptosis	^86^
		↑ mitofusion	
		↓ mitophagy	
		↓ mitochondrial complexes I and IV activity	
primary oligodendrocytes (from P6 mouse pup brain)	*p*C	impaired oligodendrocytes differentiation	^90^
		↓ myelin gene expression	
neuron2a neuroblastoma (N2a)	*p*C	↓ cell differentiation	^92^
		↓ neurite length	
pheochromocytoma (PC-12)	*p*C	↓ neurite length	^92^
rat hippocampal neurons	*p*C	↓ dendritic arborization	^92^
		↓ synaptic density	
		↓ neuronal activity	
		↓ intracellular Ca^2+^ signaling	
rat pheochromocytoma cells (PC-12)	*p*C (low doses)	↑ neurite outgrowth	^101^
		↑ NF expression	
		↑ BDNF secretion	
human brain capillary endothelium model (hCMEC/D3)	*p*C	↑ paracellular permeability	^109^
conditionally immortalized human renal proximal tubule epithelial cells (ciPTEC)	*p*C/*p*CS/*p*CG	↓ mitochondrial succinate dehydrogenase activity	^85^
splenocytes	*p*C	↓ IFN-γ production	^105^
		↑ IL-4 production	
	*p*CS	↓ IFN-γ production	
		↑ IL-4 production	
		↓ % Th1 cells and Th1/Th2 ratio	
		↑ % Th2 cells	
human proximal tubular cells (HK-2)	*p*CS	↑ ROS production	^59^
		↑ NOX activity	
		↑ mRNA levels of inflammatory cytokines	
		↑ active TGF-β1 protein secretion	
human brain capillary endothelium model (hCMEC/D3)	*p*CS	↑ BBB permeability	^64^
		↓ endothelial junction disruption	
		↑ metalloproteinases activity	
human endothelial cell line (EA.hy926)	*p*CS	↑ CREB/ATF1 signaling activation	^69^
		↑ ATF1 protein levels	
		↑ endothelial inflammation	
		↑ oxidative stress	
mouse 3T3-L1 adipocytes	*p*CS	↑ ROS production	^70^
		↑ NADPH activation	
		↓ intracellular GSH content	
human endothelial cell line (EA.hy926)	*p*CS	formation of extracellular vesicles (EVs)	^81^
human renal tubular cells (HK2)	*p*CS	aerobic/anaerobic metabolism impairment	^84^
		↓ mitochondrial mass	
		↑ autophagy	
		↑ mitochondrial fission	
primary cultures of hippocampal and cortical neurons	*p*CS	↑ neuronal dysfunction	^93^
		↓ firing rate	
		↓ spikes/sec	
		↓ network bursts/sec	
macrophage-like cell line (RAW246.7)	*p*CS	↓ IL-12 p40 production	^104^
		↑ IL-10 production	
		↓ NO production	
		↓ LPS-induced CD40 expression on the cell surface	
primary peritoneal macrophages	*p*CS	↓ IL-12 p40 production	^104^
		↑ IL-10 production	
neuroinflammation model of LPS-activated BV2 microglia	*p*CS	↓ ADAM10 and ADAM17 expression	^106^
		↓ TNF-α and IL-6 release	
		↓ constitutive microglia phagocytosis	
human brain capillary endothelium model (hCMEC/D3)	*p*CG	↑ transendothelial electrical resistance	^109^
***In Vivo***			
C57BL6/J mice	*p*C	↓ social interaction	^60^
		↑ stereotyped/perseverative behaviors	
		↓ VTA dopaminergic neurons activity microbiota remodeling	
Wistar rats	*p*C	↑ GLUN2B/GLUN2A ratio in Nac	^88^
		↓ GLUN2B/GLUN2A ratio in hippocampus	
		↓ CREB phosphorylation in Nac	
		↓ Rac1 activity in Nac	
		↑ Rac1 activity in hippocampus	
audiogenic seizure-prone Krushinski–Molodkina (KM) rats	*p*C	↓ GLUN2B/GLUN2A ratio in Nac	^88^
		↑ GLUN2B/GLUN2A ratio in hippocampus	
		↓ CREB phosphorylation in hippocampus	
		↓ Rac1 activity in Nac	
		↑ Rac1 activity in hippocampus	
inbred murine model of ASD (BTBR mice)	*p*C	↓ social interaction stereotyped/perseverative behaviors	^96^
		↑ anxiety-like behavior	
		↑ DA turnover	
Wistar rats	*p*C	↓ Na^+^/K^+^-ATPase activity	^102^
		↓ total ATPase activity	
		↑ lipid peroxidation	
5/6 nephrectomy Sprague–Dawley rats CKD model	*p*CS	↑ oxidative stress	^59^
		↑ NOX activity	
		↑ upregulation of NOX4	
		↑ renal damage	
		↑ TGF-β1 production	
unilateral nephrectomized C57BL/6 mice	*p*CS	↑ depression/anxiety-like and cognitive impairment	^62^
		↑ oxidative stress	
		↑ neuroinflammation	
		↓ serum levels of BDNF and 5-HT	
		↑ serum levels of corticosterone	
		↑ pro-inflammatory cytokines	
		↓ neurogenesis and cell survival	
		↓ GSH and MDA	
C57BL6/J mice	*p*CS	↑ BBB permeability	^64^
		↓ neuronal activity	
B-6 mice with 1/2-nephrectomy mice	*p*CS	mitochondrial function impairment	^84^
C57BL6/J mice	*p*CG	↓ BBB permeability	^109^
		↓ pathways associated with cellular degradation and metabolism	
		↑ pathways associated with growth factor/transcription factor signaling	

a ADAM: a disintegrin and metalloprotease; ATF1: activating transcription factor 1; BBB: blood–brain barrier; BDNF: brain-derived neurotrophic factor; CD40: cluster of differentiation 40; CREB: cAMP response element-binding protein; DA: dopamine; DCF: 2′-7′-dichlorofluorescein; GLUN2A: NMDAR subunit 2A; GLUN2B: NMDAR subunit 2B; GSH: glutathione; 5-HT: serotonin; IFN-γ: interferon-gamma; IL: interleukin; LDH: lactate dehydrogenase; LPS: lipopolysaccharide; MDA: malondialdehyde; Nac: Nucleus accumbens; NF: neurofilament; NO: nitric oxide; NOX:NADPH oxidase; NOX4: NADPH oxidase-4; ROS: reactive oxygen species; TGF-β1:transforming growth factor β1; TNF-α: tumor necrosis factor-alpha; and VTA: ventral tegmental area.

Among the various cellular targets affected by *p*C and its metabolites, the vascular endothelium, and, in particular, the blood-brain barrier (BBB), emerges as a critical site of vulnerability.^64–66^ *p*CS can impair BBB integrity by activating epidermal growth factor receptor (EGFR) signaling and promoting matrix metalloproteinase release, leading to extracellular matrix breakdown, cytoskeletal changes, and weakened cell junctions that increase permeability.^64^ In brain microvascular endothelial cells, these effects translate into a direct compromise of BBB integrity, allowing peripheral pro-inflammatory factors and neurotoxic metabolites to penetrate into the CNS.^67^ This loss of barrier selectivity is not only a consequence of systemic toxicity but a key amplifier of central damage, facilitating the widespread neuronal, metabolic, and immune alterations that will be discussed in the following sections.^67^

### Oxidative Stress (A)

*p*C and *p*CS contribute to cellular and tissue damage, both systemically and in the CNS, primarily by inducing oxidative stress. Evidence, including a recent systematic review, shows that these uremic toxins disrupt redox balance, weaken antioxidant defenses, and increase reactive oxygen species (ROS), causing extensive molecular and mitochondrial damage.^68^

One important mechanism by which *p*CS contributes to oxidative stress is through activation of NADPH oxidase (NOX), leading to the production of superoxide anions and other ROS.^59,69,70^ This NOX-mediated ROS generation, particularly in microglia and astrocytes, has been strongly linked to neuroinflammation and disruption of the BBB, processes involved in neurodegenerative and neurodevelopmental disorders.^68,71–73^ Similarly, *p*C metabolism via cytochrome P450 produces reactive intermediates that further enhance ROS generation.^74^ In the CNS, oxidative stress may be intensified by excess dopamine (DA), which accumulates, due to *p*C-mediated inhibition of dopamine β-hydroxylase (DBH).^75,76^ Consequently, this DA surplus undergoes spontaneous oxidation to toxic quinone derivatives and ROS that exacerbate oxidative stress.^63,77,78^ The resulting oxidative imbalance involves an increase in pro-oxidant markers and a concurrent weakening of antioxidant defenses, creating a cellular environment that is highly vulnerable to oxidative injury.^62^ This vulnerability is further exacerbated by impaired detoxification mechanisms, particularly the depletion of intracellular glutathione (GSH), a key molecule in maintaining redox homeostasis.^79^ When GSH is reduced, reactive intermediates accumulate and interact with cellular components, leading to functional impairment and structural damage.^80^ In hepatic models, *p*C, and to a lesser extent its conjugates *p*CS and *p*CG, have been shown to deplete GSH and induce necrotic cell death, as evidenced by increased lactate dehydrogenase (LDH) release.^41^ These responses reflect a compromised redox balance and damaged cellular integrity, in agreement with the effects reported in other systems.^62,70^ Moreover, *p*CS induces the release of extracellular vesicles (EVs) that carry ROS-generating enzymes, which can increase oxidative damage by propagating stress to neighboring cells and further amplifying oxidative damage.^81^

### Mitochondrial Dysfunction (B)

The increased ROS production, eventually triggered by *p*CS and *p*C, leads to significant mitochondrial damage by targeting key mitochondrial components.^82^ Specifically, *p*CS-induced ROS production, via mitochondrial NOX4 activation, inhibits complex I in the electron transport chain, impairing respiration and reducing ATP synthesis.^83^ Beyond this, *p*CS disrupts aerobic/anaerobic respiration by promoting fission and inhibiting fusion through ROS-dependent mechanisms and worsens mitochondrial dysfunction by downregulating key proteins for stability and calcium homeostasis.^84^ Together with the effects on mitochondrial dynamics, both *p*C and *p*CS also inhibit succinate dehydrogenase activity (28% and 21%, respectively), while *p*CG causes a milder reduction (14%).^85^ Additionally, *p*C downregulates respiratory chain complexes I and IV, leading to reduced ATP production and increased apoptosis.^86^ These mitochondrial impairments have been observed in various cell types;^83–87^ however, their impact on neuronal cells remains poorly explored. Due to neurons high-energy needs and sensitivity to ROS, more research is needed to assess how *p*C and its conjugates impact mitochondrial function in the CNS.

### Neuroinflammation and Damage to Neurons and Oligodendrocytes (C)

Alongside oxidative stress, *p*C and *p*CS contribute to neuroinflammation and neurodegeneration through a combination of pro-inflammatory signaling and impaired neuroplasticity, ultimately disrupting the integrity and function. In nephrectomized mice, *p*CS increases IL-1β expression and activates key inflammatory pathways (JNK, p38, NF-κB) in the prefrontal cortex (PFC), contributing to neuronal damage.^62^ These changes are accompanied by elevated caspase-3 activity and reduced MAP-2 expression, reflecting increased apoptosis and structural impairment, as well as downregulation of essential neurogenic and proliferative markers.^62^ Both *p*C and *p*CS can also significantly reduce phosphorylation of CREB, a transcription factor crucial for regulating genes involved in synaptic plasticity, neurogenesis, and neuronal survival.^62,88,89^ This impairment has been observed in various brain regions, suggesting a broad disruption of the neuronal function. Reduced CREB activity is also accompanied by decreased activation of upstream regulators, like Akt and PKA, further weakening neuroplastic responses.^62^ In addition to its effects on neurons, *p*C interferes with oligodendrocyte maturation by inhibiting the expression of myelin-associated genes and preventing the differentiation of oligodendrocyte progenitor cells into myelinating cells, with these effects being especially notable in the medial PFC.^90^ Beyond this, it is also hypothesized that *p*CS may contribute to neuroinflammation by promoting activation of the NLRP3 inflammasome, which plays a central role in triggering pro-inflammatory cytokine release and amplifying inflammatory responses.^91^ However, evidence suggests that the uremic toxin indoxyl-sulfate (IS) has a more pronounced impact on this pathway, showing a stronger association with neuroinflammatory processes and cognitive dysfunction.^91^ While *p*CS may still be involved, its connection to NLRP3 activation remains less clear, warranting further investigation to understand its precise role in neuroinflammation.

### Synaptic Alterations (D)

Guzmán-Salas *et al.*have studied *p*C effects on neuronal cells and hippocampal cultures, finding that this compound significantly reduced synaptic density by downregulating key synaptic markers (PCLO, SHANK, PSD-95, VGAT), which are vital for synapse formation and neurotransmission.^92^ Prolonged exposure to *p*C was also associated with reduced spontaneous neuronal activity and lower intracellular calcium levels, factors that may underlie the compound's proposed role in exacerbating ASD symptoms, as they compromise the functional dynamics of neural circuits.^92^ Additionally, even short-term *p*C exposure reduced neurite length for at least 24 h, while higher concentrations impaired dendritic arborization by inhibiting primary and secondary dendrite growth, changes similar to those seen in ASD.^92^ This supports the hypothesis that *p*C may play a role in abnormal neurodevelopment, consistent with earlier findings of reduced Rac activity, a protein important for dendritic growth and synaptic plasticity, following *p*C exposure.^88^ Similarly, chronic exposure to *p*CS can reduce neuronal network activity by impairing the firing rate and burst frequency, interestingly, without affecting mitochondrial function or oxidative stress.^93^ Overall, these effects support a direct role for *p*C and *p*CS in functional synaptic disruption, potentially contributing to broader neurodevelopmental and neurodegenerative disorders.

### Neurotransmitter Metabolism Dysfunction (E)

Another key neurotoxic action of *p*C is its competitive inhibition of DBH, the enzyme that converts DA into norepinephrine (NE), an essential step for maintaining catecholamine balance.^63,75,76,94^ By covalent binding to DBH, *p*C alters normal DA metabolism, leading to its accumulation and reduced levels of downstream catecholamines. Clinical studies support this mechanism, showing that individuals with ASD have elevated urinary levels of both DA and *p*C, alongside reduced NE and epinephrine (EPI), suggesting functional DBH inhibition *in vivo*.^95^ Experimental models further confirm that high *p*C exposure increases DA as well as its metabolites 3,4-dihydroxyphenylacetic acid (DOPAC) and homovanillic acid (HVA) in various brain regions, while NE levels remain unchanged.^96^ Furthermore, *p*C exposure was found to suppress the activity of dopaminergic neurons in the ventral tegmental area (VTA), reinforcing its role in dopaminergic dysregulation.^60^

In contrast, *p*CS can interfere with critical neurotrophic and monoaminergic systems. Specifically, *p*CS reduces the levels of serotonin (5-HT) and brain-derived neurotrophic factor (BDNF), which are essential for neuronal survival, plasticity, mood regulation, and cognitive functions.^62^ In the prefrontal cortex, *p*CS lowers 5-HT levels, thus affecting serotonergic transmission,^62^ a process closely linked to depression, anxiety, social dysfunction, and the cognitive decline observed in neurodegenerative diseases, including both AD and PD.^97,98^ At the same time, *p*CS downregulates BDNF, which not only leads to cognitive decline, brain atrophy, and increased risk of psychiatric disorders but also impairs serotonergic neurons themselves.^99,100^ These effects are reinforced by the upregulation of REST, a transcriptional repressor that silences genes involved in both 5-HT and BDNF pathways, worsening neuropsychiatric vulnerability.^62^ Interestingly, *p*CS may exert a dual effect depending on its concentration; mild oxidative stress induced by low *p*CS levels can transiently stimulate BDNF release and activate redox-sensitive genes, promoting neuroplasticity, whereas higher levels can cause detrimental effects.^101^

### Receptor Activity Disruption (F)

The impact of this toxin on NMDA receptors (NMDARs) was explored by Gigi *et al.*,^88^ who found that exposure to *p*C significantly altered the composition of NMDAR subunits, particularly increasing the GLUN2B/GLUN2A ratio in a region-specific manner. This effect is partly mediated by *p*C modulation of the interaction between NMDAR subunits and dopamine D1 receptors, affecting NMDAR surface expression and localization.^92^ Additionally, *p*C can affect the expression of neurofilaments (NFs), proteins that interact with both glutamatergic and D1 receptors and play a vital role in neuronal structural remodeling, further amplifying the changes induced by *p*C.^101^ Alongside these effects on receptor composition and structural proteins, *p*C is also associated with reduced activity of Na^+^/K^+^-ATPase, an essential enzyme for maintaining neuronal excitability and signaling, which may further contribute to its neurotoxic impact.^102^

### Systemic Immunosuppression (G)

The potential damage caused by *p*C and, mainly, its metabolite *p*CS, appears to be significantly related to alterations in immune responses across different cell types.^103–105^ *p*CS can suppress Th1-type cellular immune responses, a key component of the body's defense against infections and inflammation.^105^ This was demonstrated *in vivo* through hypersensitivity tests, where *p*CS exposure led to decreased IFN-γ production and increased IL-4 levels in T cells, indicating a shift toward a Th2-type immune response that weakens effective Th1 defenses.^105^ Moreover, *p*CS is able to alter macrophage functions by suppressing the production of pro-inflammatory cytokines, such as IL-12, while promoting the production of anti-inflammatory cytokines, like IL-10.^105^ This imbalance, alongside a reduction in nitric oxide (NO) production and decreased CD40 expression on macrophages, further impairs Th1-type immune responses, potentially undermining the body’s defense mechanisms.^104^

### Microglial Dysfunction and Impaired Brain Immune Responses (H)

This immunosuppressive profile of *p*CS extends to the CNS, where it directly impacts microglia, the brain resident immune cells. In BV2 microglial cells, *p*CS inhibits both constitutive and lipopolysaccharide (LPS)-induced production of pro-inflammatory cytokines (TNF-α and IL-6) by inhibiting ADAM10 and ADAM17 enzymes, which are essential for cytokine processing and release.^106^ This reduction suggests that *p*CS disrupts cytokine-mediated immune responses, potentially impairing immune signaling and contributing to neurological conditions, such as ASD, where immune dysregulation plays a key role.^106^ Additionally, increased microglial CD68 expression after *in vivo* *p*CS exposure suggests microglial involvement in *p*CS-driven neuroinflammation.^62^

### *p*CG between Detoxification and Toxicity

While the toxic effects of *p*C and, more consistently, its sulfate conjugate *p*CS have been well documented, the role of its glucuronide counterpart *p*CG remains less clearly defined. Several studies have demonstrated that *p*CG is significantly less toxic than both *p*C and *p*CS across various human cell types, including kidney, liver, and blood cells.^41,44,85,107,108^ In hepatocyte models, for example, *p*CG showed minimal cytotoxicity, even at elevated intracellular concentrations, suggesting that glucuronidation serves as an important detoxification mechanism to neutralize the harmful effects of its parent compound.^41^ In agreement with this, inhibiting *p*CG formation results in increased cellular damage, as indicated by a higher LDH release and the accumulation of free *p*C.^41^ Beyond its role in detoxification, these findings also challenge the traditional assumption that glucuronide conjugates are biologically inert and simply destined for renal excretion. Notably, *p*CG appears to work as a functional antagonist of toll-like receptor 4 (TLR4), preventing the receptor activation by bacterial endotoxins, such as LPS, thereby protecting the BBB from LPS-induced permeability.^109^ This suggests a possible neuroprotective effect against pro-inflammatory stimuli of *p*CG under physiological conditions, particularly within the context of gut-brain communication. Nevertheless, *p*CG is classified as a uremic toxin, and it has been found at elevated levels either in patients with CKD or in those undergoing hemodialysis, where it may exert harmful effects.^43,85,110^ These findings highlight the need to re-evaluate glucuronide conjugates not merely as passive metabolic byproducts but as active modulators, particularly at the level of the cerebral vasculature. The contradictory nature of the current data highlights the need for further research to fully understand its impact on human health.

## Effect of *p*-Cresol and Conjugated Metabolites on Neurodevelopment: Autism

ASD comprises a range of neurodevelopmental disorders including autistic disorder, Asperger syndrome, and pervasive developmental disorder. Typically manifesting in early childhood, this pathology is primarily characterized by deficits in social interaction and communication, as well as by restricted interests and stereotyped behavior.^111^ Clinical manifestations of ASD exhibit significant interindividual variability in terms of symptom presentation, severity, developmental trajectory, and treatment response. Over the past two decades, the incidence of ASD has increased markedly: recent estimates from the Autism and Developmental Disabilities Monitoring (ADDM) Network indicate that approximately one in every 36 children aged eight or older in the United States is diagnosed with ASD.^112^ Consequently, a condition once considered rare has emerged as one of the most prevalent disorders in child neuropsychiatry.

Both genetic and environmental factors are implicated in the pathogenesis of ASD. Recent research has strongly highlighted the role of *p*C and its conjugated metabolites in ASD.^55^ Elevated urinary levels of these compounds have been detected in the urine of a subset of ASD individuals. A study carried out in Italy by Altieri *et al.* in 2011, later replicated in France by Gabriele *et al.* in 2014, has highlighted significantly higher urinary levels of *p*C, *p*CS, and *p*CG in autistic children.^111,113^ In these studies, higher levels of total *p*C in urine were observed in ASD children,^111,113^ with particular regard to *p*CS, leading to the conclusion that *p*CS may be the real toxin under ASD onset.^113^ In addition, increased levels of *p*CS were found in females with ASD compared to males, as consistently reported across multiple studies,^55^ including those by Altieri *et al.*,^111^ Gabriele *et al.*,^113^ and more recently Osredkar *et al.*,^114^ suggesting possible sex-related differences in metabolism or microbiota composition rather than inherent severity. Nevertheless, more studies with larger and more gender-balanced cohorts are needed to verify this gender-related difference.

Other studies have pointed out that *p*CS levels are significantly higher in children with ASD children and comorbid conditions, such as epilepsy, hearing loss, developmental delays, ADHD, and tic disorders;^55^ higher urinary levels of both *p*C and *p*CS were also positively correlated with the severity of ASD symptoms.^55,114,115^ It should be pointed out that in one of the studies cited it is not clear whether the authors refer to the total *p*C level in urine or only to the unconjugated form,^115^ making it difficult to clearly state if either *p*C or *p*CS or both are correlated to the severity of ASD. It is worth noting that urinary *p*C levels are especially high in ASD subjects with chronic gastrointestinal (GI) conditions linked to gut dysbiosis and abnormal abundance of *Clostridium* species,^116^ among the main producers of this toxin. Many studies have described a correlation between ASD and increased presence of *C. difficile*, contributing to explaining the increased levels of total *p*C in these individuals.^117–119^ Specifically, an abnormal abundance of *Clostridium* species in the intestines has been correlated with increased total urinary *p*C levels in autistic children under the age of 8.^111^ Additionally, a correlation between environmental glyphosate exposure and *C. difficile* proliferation has been pointed out, suggesting that glyphosate, a common herbicide, may contribute to gut dysbiosis by promoting the overgrowth of *Clostridium* species.^120^

It has been shown that *p*C levels are also higher in the feces of autistic children compared to controls, with a negative correlation between these levels and age:^121^ this suggests that younger children with ASD may be exposed to greater concentrations of this toxin, potentially making it a useful biomarker for autism in early childhood.^106,111,113,121^ Meta-analyses were carried out on the studies correlating fecal levels of *p*C and ASD, but they were not conclusive; *p*C levels were consistently higher in the ASD group, but they did not reach a statistically significant difference.^122^

Increased total urinary *p*C concentrations have been associated with stool consistency, indicating that slow intestinal transit and chronic constipation, both common in individuals with ASD, are key factors influencing its accumulation.^116,123^ In fact, approximately 40% of children with ASD have obvious functional GI issues such as diarrhea, constipation, bloating, and abdominal pain, which may be linked to an altered GMB and the overproduction of *p*C.^55^ Further supporting this, a longitudinal study on chronically constipated autistic children (aged 2–8) has shown that accelerating intestinal transit through polyethylene glycol (PEG) administration led to a noticeable reduction in anxiety, hyperactivity, social interaction deficits, and stereotyped behaviors.^116^ This clinical improvement was accompanied by a decrease in the urinary *p*C level, although with some variability, likely due to the unpredictable effects of PEG on GMB composition.^116^ By shortening the intestinal transit time, PEG may either promote or inhibit *p*C production, depending on individual microbiome characteristics. Regardless of whether *p*C originates primarily from environmental exposure or gut bacterial metabolism, these findings highlight the crucial role of intestinal transit in determining its systemic levels, reinforcing the idea that GI health is closely linked to behavioral symptoms in ASD.^116^ It should be emphasized that the study by Turriziani *et al.* ^116^ refers to total *p*C levels in urine and they do not discriminate among *p*CS, *p*CG, and free *p*C; the increased level of *p*C in urine, therefore, cannot be attributed to a specific compound.

The causative correlation between exposure to *p*C and ASD development has been increasingly highlighted through studies showing its potential to impair brain function and behavior. It has been observed that mice exposed to *p*C for 4 weeks in drinking water presented autistic-like symptoms, including social deficits, stereotypies, and perseverative behaviors, but no changes in anxiety, locomotion, or cognition were observed.^60^ Similarly, BTBR mice, a murine model of ASD-like phenotype, displayed heightened anxiety and hyperactivity when administered a low dose of *p*C (1 mg/kg), while a higher dose (10 mg/kg) exacerbated typical ASD traits, including stereotypic behaviors and social deficits.^96^ This increase in severity was accompanied by accelerated dopaminergic turnover in the amygdala and dorsal striatum, critical areas for emotional and motor control, providing further insight into how the exposure to *p*C alters brain chemistry in a way that mirrors the primary and secondary symptoms seen in humans with ASD.^96^ In other studies, intraperitoneal injection of *p*C for 21 days considerably induces autism-like behavioral alterations in rats^124^ and increases their seizure readiness, suggesting a possibility of a common mechanism involved in the *p*C-induced development of both autism and epilepsy.^125^ Confirming a possible correlation between the two disorders, 40% of autistic children experience epilepsy.^125^ Behavioral abnormalities were paralleled by neurochemical alterations in mice, mainly involving the dopaminergic turnover.^96^ This interpretation aligns with long-standing evidence of DBH inhibition exerted by *p*C and with the proportionate increase in DA and its metabolites, supporting increased DA accumulation, release, and catabolism (intra- and extra-cellular).^96^ This has been proposed as a possible cause of the social and sensory issues observed in individuals with ASD.^112^ Nevertheless, in all these studies it was not investigated whether *p*C itself or its metabolites was responsible for the effects.^60,96,124,125^ A study by Vinithakumari *et al.*, involving C57BL6/J mice treated with a mixture of antibiotics and *C. difficile* spores, has revealed neurotoxic effects after the induction of *C. difficile* infection.^94^ These mice developed symptoms like diarrhea, weight loss, and increased DA levels in the nigrostriatal pathway.^94^ Despite the elevated DA, a significant decrease in hippocampal NE was also observed, impairing their ability to consolidate social memories.^94^ The authors observed that *C. difficile* infection leads to an increase in serum and intestinal *p*C levels, which could exert its effects on neurotransmission after crossing the BBB.^94^

All of these findings suggest that *p*C has a profound impact on both neurotransmitter systems and the neural circuits that regulate behavior and cognition, suggesting that it may interfere with the neural circuits involved in phenotypical core aspects of ASD.

These biochemical changes, observed in murine models, are not limited to animals, as human studies mirror these findings. Indeed, abnormal levels of DA and NE have been observed in autistic children, strengthening the connection between neurotransmitter imbalances and ASD.^95^ The analysis of urine samples from 40 autistic children has shown significantly higher levels of DA and HVA, coupled with lower levels of NE.^95^ The cause of this neurotransmitter imbalance is thought to be linked to the ability of *p*C to inhibit DBH, as previously described in the “Mechanisms of damage caused by *p*-cresol and metabolites” section. This accumulation, particularly in the form of HVA, has been strongly correlated with the severity of ASD symptoms, including heightened agitation and stereotypic behaviors.^95^

The existing studies on autism, taken together, strongly suggest that *p*C and its metabolites may play a crucial role in the pathogenesis of ASD. Human studies showed elevated urine levels of *p*C and its metabolites in certain ASD patients, particularly those with chronic GI issues linked to dysbiosis, indicating that these compounds could play a significant role in the development and progression of the disorder.^122^ A very recent meta-analysis on urine studies highlighted that the levels of *p*C and its conjugated metabolites could be a potential biomarker for ASD, also considering its strict link to the increased relative abundance of specific intestinal bacteria.^122^

The mechanism through which *p*C and its conjugated metabolites exert their detrimental effects are likely to be related to their ability to boost oxidative stress in several districts, comprising brain tissues, and to alter the neurotransmitter system,^68^ as described in the dedicated section of this review. Collectively, these effects exacerbate the pathophysiological onset, progression, and severity of a broad spectrum of diseases, including neurodevelopmental conditions, such as ASD.^68^ Their occurrence in specific brain tissues could be due to the disruption of the BBB or mediated by OATs.^68^

The presence of *p*C in autistic children, specifically produced by *C. difficile*, in an altered GMB scenario, further supports the concept of a gut-brain axis and offers new insights into possible therapeutic intervention, both pharmacological and nutritional.^95^

Unfortunately, the available literature often does not specify or investigate whether the observed effects are exerted by unconjugated *p*C or by its conjugated metabolites, making it difficult to speculate which is most responsible for neurotoxicity. Nevertheless, recent findings emphasize the importance of considering environmental factors, such as *p*C exposure, when investigating the broader causes of ASD.

## *p*-Cresol and Conjugated Metabolites in Parkinson’s Disease

PD is a progressive neurodegenerative disorder that symptomatically evolves through a combination of motor symptoms, such as tremors and bradykinesia, and nonmotor symptoms, including GI dysfunctions.^126^ Growing evidence indicates that PD can be viewed as a multifactorial disorder, with its onset and progression likely driven by an interplay of age, genetic factors, and environmental influences.^127,128^

Etiopathogenesis of PD, notably the formation of Lewy bodies and the α-synuclein pathology, has been linked to the enteric nervous system, with emerging evidence suggesting that these structures may spread to the brain, further propagating the disease via cell-to-cell transmission.^129,130^ This gut-brain connection is further supported by clinical observations that GI symptoms often precede motor impairments by years, highlighting a potential role for the GMB both at the onset and throughout the course of the disease.^131,132^ Increasing evidence suggests that GI comorbidities, such as constipation, delayed colonic transit, and small intestinal bacterial overgrowth, are highly prevalent in PD patients.^133–139^ Among the many functions of the GMB, its role in metabolism is particularly relevant to PD, as it aids in the breakdown of dietary components, fermenting carbohydrates into short-chain fatty acids (SCFAs) and proteins into aromatic amino acid (AAA) metabolites.^140^ These metabolites, including l-phenylalanine, l-tyrosine, and l-tryptophan, are involved in various physiological processes that regulate immune, inflammatory, metabolic, and neuronal responses in the gut and the brain. The dysregulation of these metabolic pathways is thought to contribute to the pathophysiology of neurodegenerative diseases, including PD.^141,142,153^ Imbalanced GMB may help explain the elevated levels of *p*C and its metabolites observed in PD patients, with these changes mainly attributed to bacteria from the *Lactobacillaceae* and *Bifidobacteriaceae* families.^143^ Increased gut permeability, a feature also described in chronic CKD, further facilitates the circulation of uremic toxins and has similarly been reported in PD patients.^144^ Environmental exposures to pesticides (such as paraquat and rotenone) and pollution have also been increasingly linked to PD,^145–148^ suggesting that microbial byproducts, including *p*C and its derivatives, are potentially influenced by these exposures and they may play a significant role in the pathogenesis of the disease.

In light of this, several studies have sought to deepen our understanding of how the GMB and its metabolites may contribute to PD. Cirstea *et al.* ^149^ recently provided evidence linking altered gut microbial metabolism, disturbed gut function, and PD. Compared to healthy controls, PD patients exhibited a reduction in butyrate-producing taxa, alongside an increase in bacterial groups linked to *p*C synthesis. These microbial shifts were reflected in increased serum levels of *p*C, *p*CS, and phenylacetyl-l-glutamine, a metabolite derived from microbial processing of phenylalanine, which positively correlated with PD status as well as the severity of GI dysfunction, firmer stools, and constipation severity.^162^

Untargeted metabolomic analyses of serum and plasma further supported the role of *p*C and its derivatives in PD-associated metabolic disturbances.^25,150,151^ Elevated levels of *p*C were observed only in serum, whereas increased *p*CS and *p*CG were detected in both serum and plasma of PD patients. The serum analysis also revealed a positive correlation between *p*C and its metabolites and age across both PD patients and controls, suggesting that aging might influence the metabolism or accumulation of these compounds. Interestingly, previous studies also reported that *p*CS levels do not correlate with its metabolic precursor, l-tyrosine, further supporting the idea that its accumulation may depend more on age-related changes than on precursor availability.^152^ On the contrary, this age-related correlation was not observed in plasma.^150^

Notably, circulating *p*CS levels across studies were strongly associated with GI disturbances, such as constipation, but they were not related to motor impairments, indicating that these microbial metabolites may primarily contribute to GI dysfunction in PD.^147,149^ In contrast, *p*CG was consistently elevated in plasma and serum of PD patients and it positively correlated with motor symptom severity. This supports a potential link between *p*CG accumulation and the progression of motor symptoms in PD.^150,151^

Beyond peripheral circulation, two recent studies have described elevated levels of *p*CS also in the CSF of PD patients compared to controls.^56,154^ Targeted metabolomics revealed that CSF levels of *p*CS in PD patients were approximately eight times higher than those in plasma, suggesting that individuals with PD accumulate more *p*CS in the brain than their healthy counterparts.^56^ One likely contributor to the increased entry of potentially neurotoxic microbial metabolites into the brain is a compromised BBB, which is known to be more permeable in PD patients.^155,156^ In addition to passive diffusion through a more permeable BBB observed in PD patients, Sankowski *et al.* suggested that specific transport mechanisms might facilitate *p*CS entry into the brain.^56^ OATs, particularly OAT3, are believed to be involved in this process. While primarily known for renal tubular secretion, some OAT isoforms, including OAT3, are also expressed in the brain, where they regulate metabolite exchange between the blood, cerebrospinal fluid, and brain tissue.^157,158^ Notably, OAT3 has been implicated in the clearance of other uremic toxins in the brain, such as IS, making it a likely candidate for mediating *p*CS transport as well.^159,160^ The disproportionate CSF-to-plasma ratio of *p*CS in PD patients may reflect a potential dysfunction in its distribution, potentially resulting from impaired OAT3 activity. This dysregulation could compromise the efflux of *p*CS from the CSF to the blood, thereby possibly contributing to its accumulation within the CNS.^56^ Nevertheless, the role of OATs in mediating *p*CS transport across the BBB has not yet been directly investigated in the context of PD, and no studies have so far demonstrated altered expression or activity of these transporters in PD patients; their proposed involvement thus remains a hypothesis by the authors. Moreover, the significant correlation observed between *p*CS levels in plasma and CSF suggests that the systemic accumulation of this metabolite may influence its central concentration. This finding, together with the markedly elevated CSF-to-plasma *p*CS ratio described in PD patients, points to possible impairments in its distribution or clearance at the brain-blood interface.^56^ Notably, this imbalance was also associated with reduced kidney function, as indicated by lower estimated glomerular filtration rate (eGFR), suggesting that compromised renal clearance may increase circulating *p*CS levels and thereby promote its accumulation in the CNS. Taken together, these observations support a model in which both systemic (renal) and central (BBB and transporter-mediated) mechanisms may contribute to elevated levels of *p*CS in the PD brain.

Elevated *p*CS concentrations in CSF have also been detected in metabolomic-metallomic studies of PD patients, supporting the role of this metabolite in PD pathogenesis and progression.^161^ In PD, *p*CS accumulation appears to positively correlated with metallomic disturbances, especially involving copper and iron, metals known to contribute to oxidative stress and neurotoxicity. Moreover, elevated *p*CS levels were observed in adults with high plasma lead concentrations and reduced cognitive performance, suggesting that *p*CS could also be involved in lead-associated cognitive decline and broader aging-related processes such as those regulated by longevity-regulating pathways.

In summary, gut-derived metabolites, including *p*C, *p*CS, and *p*CG, eventually influenced by environmental exposure, are increasingly recognized as key players in PD, linking microbial dysbiosis, age-related changes, renal function, and BBB integrity to both motor and nonmotor symptoms. These effects may be mediated by mechanisms previously described, involving oxidative stress, mitochondrial dysfunction, neuroinflammation, and impairment of the BBB. While their exact mechanisms and transport pathways, such as the potential involvement of OAT3, remain to be fully clarified, these findings highlight the importance of the gut-brain axis in PD pathogenesis and open new avenues for therapeutic intervention targeting microbiome-related pathways.

## *p*-Cresol and Conjugated Metabolites in Alzheimer’s Disease

AD is the most common form of neurodegenerative disorder in the elderly and the leading cause of dementia with few effective treatments currently available. The disease is characterized by pathological features, including amyloid-β-peptide (Aβ) plaques, tau protein neurofibrillary tangles, and neuroinflammation, all of which contribute to neurodegeneration. Increasing evidence points to a pivotal role of neuroinflammation in the pathogenesis and progression of AD.^163–165^ It is now known that the GMB plays a pivotal role in regulating neuroinflammation in various neurological conditions, including AD.^58,166–169^

Recent studies indicate that patients with AD exhibit an altered GMB composition compared to individuals without AD,^168,170,171^ and experimental manipulations of the GMB in mouse models have demonstrated its capacity to influence AD-related pathology and neuroinflammation.^172,173^

Even if the exact mechanisms linking GMB alterations to AD progression remain unclear, modifications of GMB composition can affect microbial-derived metabolites, which eventually modulate CNS immune responses in neurological diseases, including AD. In an AD mouse model, it has been observed that GMB dysbiosis is required to infiltrate peripheral immune cells into the brain.^174^ Once they enter the CNS, immune cells contribute to neuroinflammation and associated cognitive deficits. On the other hand, restoring GMB composition in APP/PS1 mice, a transgenic model of AD, mitigates neuroinflammation and reverses cognitive impairments.^174^

Evidence suggests a heightened risk of developing AD and other neurological conditions among patients with CKD,^175–177^ possibly due to the accumulation of protein-bound uremic toxins, such as *p*CS and IS.^178^ These toxins, primarily derived from gut microbial metabolism, are known to contribute to cognitive decline and neurodegenerative processes.^62,179^ On the other hand, the treatment with AST-120, an oral spherical activated carbon that acts as a uremic toxin adsorbent, resulted in protection against cognitive and emotional impairment in CKD patients,^179,180^ thus confirming the involvement of these molecules in impaired CNS functioning.

Overall, gut dysbiosis, involved in both CKD and AD conditions, exacerbates the production of uremic toxins like *p*CS, which, in turn, are known to increase vascular permeability, including that of the BBB.^64,65,181,182^ This results in higher levels of uremic toxins not only in the periphery (i.e., serum/plasma) but also in the brain,^91^ where these toxins can induce oxidative stress, mitochondrial dysfunction, and neuroinflammation, hallmark features of AD.^84,183,184^

The work performed by Sun *et al.* has further highlighted the potential role of *p*CS in the pathogenesis of AD.^62^ *In vivo* behavioral analyses revealed that mice exposed to *p*CS developed anxiety- and depressive-like behaviors, symptoms frequently observed in AD. Moreover, exogenous administration of *p*CS induced notable cognitive impairments, alongside increased oxidative stress, neuroinflammation, and reduced levels of BDNF and 5-HT, all of which are hallmark features of neurodegenerative diseases.^62^ Consistently, clinical studies have described decreased serum levels of both BDNF and 5-HT in AD patients,^185,186^ further supporting a mechanistic link between *p*CS exposure and neurodegenerative pathologies. Despite the limited number of studies focusing specifically on the relationship between *p*C and AD, metabolomic analyses have consistently shown elevated levels of its primary derivatives, *p*CS and *p*CG, in AD patients.^187,188^ A targeted metabolomic analysis of AD plasma and brain tissues performed by Kalecký *et al.* in nonhispanic whites has revealed notable changes in small molecules linked to GMB activity, reinforcing the critical role of the gut-brain axis in disease progression.^187^ Among these metabolites, elevated levels of *p*CS were found in plasma and approached statistical significance in the frontal cortex.^187^

Similarly, Gordon et al. have found that higher *p*CG levels were strongly associated with adverse brain aging and cognitive decline in AD patients.^188^ This was further supported by a cross-sectional study carried out in Japan, that showed increased fecal *p*C levels in dementia patients compared to those without dementia (57.5 μg/g vs 0.29 μg/g), thus reinforcing the growing evidence for the role of this compound and its metabolites in neurodegenerative diseases.^189^

Overall, emerging evidence supports a potential link between gut-derived uremic toxins, particularly *p*C and its metabolites, and the pathogenesis of AD. *p*CS and *p*CG, generated by gut microbial fermentation, accumulate in circulation under conditions such as gut dysbiosis and CKD, both linked to AD. Mechanistically, they increase BBB permeability, allowing their entry into the brain, where they trigger synaptic impairment and cognitive decline. Elevated *p*CS and *p*CG levels in AD patients further support their involvement, highlighting the gut–brain axis as a potential therapeutic target in AD.

Despite these findings, the relationship between *p*C and AD remains less thoroughly investigated than its correlation to ASD and PD, highlighting a gap in the current research.

## Effect of *p*-Cresol And Conjugated Metabolites on Post-Traumatic Stress Disorder

PTSD is a trauma- and stressor-related disorder characterized by symptoms, such as intrusion, avoidance, negative alterations in cognition and mood, and hyperarousal, as defined by the Diagnostic and Statistical Manual of Mental Disorders.^190^ These symptoms significantly affect the quality of life and often persist long after a traumatic event. While much of the early research on PTSD focused on psychological and neurological mechanisms, recent advances highlight the role of the GMB axis in mediating the disorder.^191,192^ Mendelian randomization studies in humans have demonstrated a causal association between specific bacterial taxa and PTSD risk.^193^ Protective taxa, such as *Porphyromonadaceae* and *Veillonellaceae*, contrast with others like *Phascolarctobacterium* and *Ruminococcaceae*.^193^ Malan-Muller et al. have identified a consortium of bacterial genera associated with PTSD severity, suggesting that GMB dysbiosis may exacerbate trauma-related symptoms.^191^

The interplay between GMB and mental health, with regard to PTSD, has emerged as a critical field of investigation.^194^ Indeed, GMB plays a key role in understanding the pathophysiology of PTSD and related neuropsychiatric conditions.^195^ Several studies underscore the critical role of GMB in regulating stress responses, trauma recovery, and neuropsychiatric health.^196–198^ The findings of these studies suggest that microbial alterations may contribute to the onset and persistence of PTSD. In particular, GMB can influence inflammation, stress responses, and neurotransmitter signaling, thus contributing to PTSD onset; in addition, microbial metabolites, immune system activation, and the vague nerve may represent the mechanism through which the bidirectional communication between the gut and brain is implemented.^194^ The interplay between the microbiome and the host autonomic, neuroendocrine, and immune systems may impact stress resilience and susceptibility to trauma- and stressor-related disorders, including PTSD.^199^

Recent research has focused on the role of microbial metabolites, such as *p*C, in oligodendrocyte differentiation and myelination, which represent critical processes for maintaining neural connectivity and behavioral stability.^193^ This interaction underscores the microbiota’s influence on brain regions, such as the PFC, which is pivotal for higher-order cognitive functions and emotional regulation.

In a PTSD preclinical mouse model, it has been demonstrated that changes in gut microbial composition may influence neuropsychiatric outcomes, including resilience or susceptibility to stress.^57^ Alterations in GMB, whether pre-existing or induced by trauma, can increase trauma susceptibility by enhancing the production of certain metabolites such as *p*C.^57^ These metabolites can influence the dopaminergic system, in a manner that varies depending on the brain region. A study by Laudani *et al.*, using the arousal-based individual screening model, has provided evidence for pretrauma and post-trauma GMB alterations in susceptible mice exhibiting persistent PTSD-related phenotypes.^57^ A more in-depth analysis revealed an increased abundance of pro-inflammatory bacteria (*Ruminococcaceae* and *Lachnospiraceae*) affecting brain processes, including myelination, and brain systems such as the dopaminergic neurotransmission.^57^ Whether these alterations in GMB composition could be associated with abnormal levels of metabolites that induce dopaminergic dysfunctions was investigated in the study of Laudani *et al.* ^57^ High levels of *p*C were detected exclusively in the PFC of susceptible mice.^57^ In addition, abnormal levels of DA and DOPAC were observed, together with a detrimental increase of D3 receptor expression in the same area.^57^ Conversely, either resilience mechanisms aimed at counteracting these *p*C-induced dopaminergic dysfunctions or myelination-related resilience mechanisms were observed only in the PFC of resilient mice.^57^ These findings are in agreement with previous evidence on *p*C, which was demonstrated to be able to affect myelination by blocking myelin gene expression and differentiation^90^ and to affect the dopaminergic system.^76^

PTSD is associated with significant alterations not only in GMB composition but also in immune system function. A work carried out by Petakh *et al.* has pointed out the interaction between PTSD, gut dysbiosis, and elevated inflammatory biomarkers, suggesting that microbial metabolites modulate immune and brain functions in PTSD.^200^

*p*CS has also been linked with behavioral disorders and neuroinflammation.^62^ In a study by Sun et al. on unilateral nephrectomized mice, it was observed that, after administration of the protein-bound uremic toxin *p*CS at a dose of 100 mg/kg/day, the serum *p*CS concentration was progressively increased; in particular, there was an accumulation of *p*CS in the PFC tissues, and this was associated with several behavioral disorders, such as depression, anxiety, and cognitive impairment.^62^ In addition, the authors have demonstrated that *p*CS exacerbates oxidative stress and neuroinflammation in CNS.^62^ It should be pointed out that this study relied on the administration of a supraphysiological dose of *p*CS and that the end points were not focused on PTSD onset. Nevertheless, these findings suggest that *p*CS and similar neurotoxic metabolites further complicate the gut-brain axis in chronic conditions and they may contribute to neurodegenerative processes observed in PTSD.^62^

The damages at the brain level exerted by *p*C and *p*CS could be explained by their capacity to cross BBB by various mechanisms.^68^ Even if only the study by Laudani *et al.* ^57^ to date has demonstrated a possible link between the presence of *p*C in specific brain regions and the onset of typical PTSD symptoms, this study has opened new perspectives for investigation and potential therapeutic interventions. Clearly, further research is needed to confirm these results and to elucidate the mechanisms through which *p*C may contribute to neuropathological processes in the context of PTSD.

The therapeutic implications of targeting GMB in PTSD are relevant. Strategies based on probiotics, prebiotics, and dietary modifications can potentially modulate gut dysbiosis, thus reducing inflammation and potentially alleviating psychiatric symptoms.^194^

## Therapeutic Interventions

Given the neurotoxic role of *p*C, a reduction in its central concentration could help mitigate the molecular, cellular, and, consequently, behavioral effects associated with this metabolite.^62,193,201^ This goal could be achieved through various strategies, including modifying the composition of the GMB (i.e., fecal transplantation, prebiotics and/or probiotics administration, and diet) or modulating its distribution and elimination.

In nephrectomized mice, an animal model of impaired kidney functionality, central *p*CS accumulation has been associated with depressive-like and anxiety-like behavior and cognitive impairments.^62^ These effects were alleviated by the administration of the uremic toxin adsorbent AST-120, which also reduces neuroinflammation, oxidative stress, apoptosis, and the negative impact on neurogenesis and neurotrophic support induced by *p*CS.^62^ Clinically, AST-120 is used to slow the progression of kidney dysfunction in CKD due to its ability to bind uremic toxins like *p*C and IS. Recently, it has been demonstrated that the administration of AST-120 is also effective in improving cognitive and emotional functions in CKD animal models.^179^ Although these effects may be related to the reduction of other uremic toxins, mainly IS, it has been shown that an AST-120 treatment can prevent CKD-induced hippocampal damage (i.e., gliosis and impaired plasticity) and *p*CS accumulation within the hippocampus.^202,203^

Another strategy to modify systemic and central pC levels and related metabolites could be fecal microbiota transplantation (FMT). Indeed, FMT has emerged as a potential therapy for several diseases associated with dysbiotic GMB, including neurodevelopmental and neurodegenerative diseases.^204^ In recurrent patients suffering from *C. difficile* infection (rCDI), FMT rapidly increases urinary levels of *p*CS, *p*CG, and fecal *p*C levels.^5^ Data from an animal model also confirm that *p*C biogenesis is more responsive to FMT treatment than other microbial metabolic pathways. These findings not only suggest that these metabolites can serve as reliable markers of the response to FMT treatment but also indicate that the effectiveness of these therapies may depend on their enhanced elimination.^5^

In patients treated with *Lactobcillus gasseri* CP2305, the fecal levels of *p*C significantly decreased compared to the control group, and this effect is paralleled by an improvement of intestinal functionality. Moreover, it is interesting to note that treatment with CP2305 has also been shown to improve sleep quality in adults with mild to moderate stress^205^ and to inhibit the effects on food intake in a subchronic and mild social defeat stress mouse model.^206^

Finally, a strategy to limit *p*CS toxicity may involve the modulation of OAT, as they are involved in the uptake and elimination of uremic toxins, including *p*CS.^157^ They work by mediating the active transport of organic anions from the bloodstream into cells, where they can be further metabolized or excreted through the kidneys.^157,207^ Specifically, *p*CS represents a substrate for OATs, being preferentially recognized by OAT1 and OAT3, as demonstrated using *in vitro* and *ex vivo* models.^51,206^

The relationship between the OAT transporters and *p*CS highlights an important aspect of kidney disease management. Understanding the mechanisms behind *p*CS transport and accumulation can provide insights into potential therapeutic strategies for patients with CKD. For example, modulating the activity of the OAT transporter or developing inhibitors of *p*CS production might offer novel ways to alleviate uremic toxicity and improve patient outcomes. Additionally, the study of the transporters of the OAT in relation to *p*CS can be crucial for developing new pharmacological treatments to enhance the clearance of uremic toxins. These interventions could help improve the quality of life for individuals with impaired renal function, especially those undergoing dialysis.

Moreover, the involvement of OAT transporters in regulating the influx and efflux of uremic toxins, such as *p*CS, across the BBB is of significant interest.^160,208^ If OAT transporters in the brain, particularly OAT1 and OAT3 are impaired, whether due to kidney dysfunction or other factors, *p*CS could accumulate within the brain, exacerbating its neurotoxic effects and contributing to cognitive and mood disorders.^56^ Further research is necessary and, aimed at investigating potential therapeutic strategies to enhance the function of the OAT transporter and to mitigate the detrimental effects of *p*C and its metabolites in the CNS, thus reducing the neurotoxic burden of this compound and improving cognitive function and mental health in individuals affected by the neurological pathologies described in this review. Specifically, molecular dynamics simulations can reveal the conformational changes of the transporters and identify the interactions between the substrates and the protein during the binding, translocation in the transporter cavity, and release of the substrate on the other side of the membrane.^209^ By combining the proteomics of OATs together with computational drug discovery, the rational design of transporter-modulating compounds could be successfully achieved.

These approaches could help reduce systemic and central levels of *p*C and may represent effective therapeutic strategies to improve cognitive and emotional functions in patients with neurodevelopmental or neurodegenerative disorders associated with this compound.

## Critical Appraisal of the Literature

Although there is increasing evidence on the relevance of *p*C and its conjugated metabolites (*p*CS and *p*CG) in the onset of different neurodevelopmental, neurodegenerative, and neuropsychiatric disorders, several limitations of the cited studies are worth mentioning, in order to have a critical view on the reported results.

First, several studies have described the detection of free *p*C in plasma or serum; however, the accuracy of these findings has been increasingly questioned, due to significant methodological limitations. Indeed, many studies do not rely on an analytical methodology capable of discriminating between *p*C itself and its conjugated metabolites. Furthermore, analytical artifacts, due to the spontaneous hydrolysis of *p*CS into *p*C during sample preparation, should be considered, leading to a possible misinterpretation. These methodological issues can give the false impression that free *p*C is present in the samples analyzed. This aspect is crucial given the fact that *p*C undergoes massive sulfation, limiting the effective amount of circulating unconjugated compound. These insights highlight the need for appropriate analytical methods and support the use of validated, highly specific analytical platforms, particularly HPLC-MS/MS assays with targeted detection of conjugated forms. In addition, frequently authors do not provide any quantitative data on average concentrations detected, making a comparison of the studies and of the results almost impossible.

As to the *in vitro* and *in vivo* mechanistic studies, the main limitations are related to either a supraphysiological exposure, far exceeding expected systemic levels, or to an inappropriate administration route selected by the authors (i.e., intraperitoneal). Even if these models shed light on cellular and molecular pathways, they do not accurately mirror the typical exposure profiles in humans.

For what concerns studies on humans, the possibility to make conclusions either on sex-related differences or on the correlation between the toxin level and the severity of the disease is limited by the unbalance of the cohorts considered and by the small number of studies described in the literature, as in the case of ASD. For other pathologies, such as AD and PTSD, the number of studies investigating the role of *p*C and conjugated metabolites is still very limited, and therefore, a causation-effect correlation is still preliminary.

## Conclusions

An increasing number of studies strongly support the role of *p*C and its primary human metabolites (*p*CS and *p*CG), in the development and progression of neurodevelopmental and neurodegenerative disorders. These multifactorial conditions share common features, including the involvement of environmental risk factors and GMB alterations, which are often characterized by dysbiosis. Indeed, *p*CS is considered a biomarker of dysbiosis and it may act as a key mediator of damage within the gut–brain axis. Elevated levels of this metabolite, detected both in the periphery and in the CFS, have been reported in several CNS diseases. Both *p*CS and *p*C have been shown to induce cellular damage in neuronal and glial cells.

Mechanistically, oxidative stress, mitochondrial dysfunction, and altered inflammatory signaling triggered by *p*C may contribute to its detrimental effects on CNS functioning, impairing cell differentiation, survival, and brain plasticity.

Further studies are needed to better elucidate causal relationships, rather than merely correlative links, between *p*C and the pathogenesis of brain disorders. These insights could significantly advance the development of preventive and therapeutic strategies, including lifestyle interventions and targeted approaches to restore eubiosis, aiming to mitigate or even prevent the progression of these complex and highly prevalent and debilitating diseases.

## Abbreviations

AAA: aromatic amino acid
Aβ: amyloid-β peptide
AD: Alzheimer’s disease
ADAM: a disintegrin and metalloprotease
ADDM: autism and developmental disabilities monitoring
ADHD: attention-deficit/hyperactivity disorder
ADME: absorption-distribution-metabolism-excretion
Akt: protein kinase B
ASD: autism spectrum disorder
ATF1: activating transcription factor 1
ATP: adenosine triphosphate
BBB: blood–brain barrier
BDNF: brain-derived neurotrophic factor
CD40: cluster of differentiation 40
CKD: chronic kidney disease
CNS: central nervous system
CREB: cAMP response element-binding protein
CSF: cerebrospinal fluid
D1: dopamine receptor D1
D3: dopamine receptor D3
DA: dopamine
DBH: dopamine β-hydroxylase
DCF: 2′-7′-dichlorofluorescein;
DOPAC: 3,4-dihydroxyphenylacetic acid
eGFR: estimated glomerular filtration rate
EGFR: epidermal growth factor receptor
EPI: epinephrine
EVs: extracellular vesicles
FMT: fecal microbiota transplantation
GI: gastrointestinal
GLUN2A: NMDAR subunit 2A
GLUN2B: NMDAR subunit 2B
GMB: gut microbiome
GSH: glutathione
HpdBCA: 4-hydroxyphenylacetate decarboxylase
HPLC–MS/MS: high-performance liquid chromatography-tandem mass spectrometry 5-HT, serotonin
HVA: homovanillic acid
IFN-γ: interferon-gamma
IL: interleukin
IS: indoxyl-sulfate
JNK: c-Jun N-terminal kinase
LDH: lactate dehydrogenase
LPS: lipopolysaccharide
MAP-2: microtubule-associated protein 2
MDA: malonyldialdehyde
Nac: nucleus accumbens
NE: norepinephrine
NF: neurofilament
NF-κB: nuclear factor kappa B
NMDA: *N*-methyl-d-aspartate
NMDAR: *N*-methyl-d-aspartate receptor
NLRP3: NLR family pyrin domain containing 3
NO: nitric oxide
NOX: NADPH oxidase
NOX4: NADPH oxidase-4
OAT: organic anion transporter
p38: p38 mitogen-activated protein kinases
*p*C: *p*-cresol
*p*CG: *p*-cresol glucuronide
PCLO: piccolo
*p*CS: *p*-cresyl sulfate
PD: Parkinson’s disease
PEG: polyethylene glycol
PFC: prefrontal cortex
*p*HPA: *p*-hydroxyphenylacetic acid
PKA: protein kinase A
PSD-95: postsynaptic density protein 95
PTSD: post-traumatic stress disorder
REST: repressor element-1 silencing transcription factor
ROS: reactive oxygen species
SULT1A1: sulfotransferase-1A
SCFAs: short-chain fatty acids
SHANK: SH3 and multiple ankyrin repeat domains protein
TGF-β1: transforming growth factor β1
TLR4: toll-like receptor 4
TNF-α: tumor necrosis factor-alpha
UGT: UDP-glucuronosyltransferase
VGAT: vesicular GABA transporter
VTA: ventral tegmental area
